# 

**DOI:** 10.1038/sj.bjc.6600402

**Published:** 2002-01-02


British Journal of Cancer (2002) 86(Suppl 1), S1–S12
© 2002 Cancer Research UK All rights reserved 0007–0920/02 $25.00
www.bjcancer.com

### CT1

### THE MRC MYELOMA VII TRIAL OF STANDARD VERSUS INTENSIVE TREATMENT IN PATIENTS <65 YEARS OF AGE WITH MULTIPLE MYELOMA

*Peter Selby[1], Tony Child[1], Faith Davies[1], Kim Hawkins[2], Julia Brown[2], Sue Bell[2], Gareth Morgan[1]
[1]Academic Unit of Haematology and Oncology, University of Leeds.
[2]Northern and Yorkshire Clinical Trials and Research Unit (NYCTRU), University of Leeds.

Intensive, high dose therapy with melphalan introduced by McElwain and colleagues in 1981 yields high remission rates but evidence on survival benefits has been inconclusive. We have completed a prospective randomised Phase III trial of this approach. Standard treatment consisted of ABCM for plateau induction with α-IFN maintenance. Intensive treatment was a three-phase regimen of C-VAMP, high dose melphalan (with autologous bone marrow/peripheral blood stem cell support as appropriate) and α-IFN maintenance. 403 patients entered the trial from 82 centres. The trial was conducted according to MRC Guidelines for Good Clinical Practice in Clinical Trials.

The primary endpoints were survival and progression-free survival. Secondary endpoints included response to treatment, quality of life, cause of death and toxicity of treatment. Survival analysis was carried out with a median follow-up of 41 months for survivors. There is improved survival in the Intensive treatment group (median survival 54.1 months compared with 42.3 months in the Standard treatment group, p=0.047 log-rank test, p=0.034 Wilcoxon test). The presentation will present the results of overall survival and progression-free survival analyses and exploratory analyses of the impact of β2-microglobulin levels on treatment effect and survival time.

### CT2

### GEMCITABINE/CARBOPLATIN (GC) VERSUS MITOMYCIN/IFOSFAMIDE/ CISPLATIN (MIP): A RANDOMISED COMPARISON IN PATIENTS WITH ADVANCED NON-SMALL CELL LUNG CANCER (NSCLC)

*NH Gower[1], RM Rudd[2], LE James[1], W Gregory[1], T Eisen[3], SM Lee[1], PG Harper[4], SG Spiro[1]. [1]University College London, [2]St Bartholomew's Hospital, London, [3]Royal Marsden Hospital, [4]Guy's Hospital, London: On behalf of the London Lung Cancer Group.

In this multicentre randomised trial we have compared a regimen commonly used in Europe, MIP, with the combination GC to test the hypothesis that, in patients with advanced NSCLC, GC would be at least as effective in terms of survival but associated with better quality of life (qol) and fewer hospital admissions.

Both regimens were given in a 21-day cycle for up to 4 courses. GC comprised G 1,200mg/m$^2$ (IV days 1 and 8) and C AUC 5 (IV day 1). MIP comprised M 6mg/m$^2$, I 3G/m$^2$ and P 50mg/m$^2$ (all IV on day 1).

Between February 1999 and August 2001 a total of 422 patients were randomised (GC - 212, MIP - 210). Patient characteristics: median age 62 years, 70% male, 41% squamous cell, 47%/52% stage IIIb/IV, ECOG PS 0:25%, 1:62%, 2:11%, 3:2%. By October 2001 88%/91% (GC/MIP) patients have completed chemotherapy. Of these 64% GC and 60% MIP received the intended 4 courses. More courses were delayed (11% vs 7%, p=0.009) with GC. Haematological toxicity was the main reason for delay in both arms (60% of delays GC, 50% MIP).

GC required fewer in-patient admissions for administration (GC 14% of courses, MIP 89%) and was associated with less nausea, vomiting, alopecia (p<0.0001) and grade 3/4 constipation (p=0.02). Thrombocytopenia grade 3/4 was greater with GC (GC 8% of courses, MIP 3%, p<0.0001), but this was not associated with increased hospital admission or toxic deaths. Overall response rates were similar 37%/ 40% (GC/MIP). However, there was a survival advantage to GC, log-rank p=0.0043 and median survival was GC 10.0 months, MIP 6.5 months.

These preliminary results suggest that GC is better tolerated and associated with longer survival than MIP. Qol analyses and updated results will be presented.

### CT3

### META-ANALYSIS OF ADJUVANT CHEMORADIOTHERAPY AND CHEMOTHERAPY FOR RESECTABLE PANCREATIC CANCER INCLUDING THE FINAL RESULTS OF THE ESPAC-1 TRIAL

JP Neoptolemos*[1], DD Stocken[2], JA Dunn[2], KE Bakkevold[3], J Jeekel[4], HO Douglass[5]
[1] Dept Surgery, Royal Liverpool University Hospital UK, [2] Cancer Research UK Trials Unit, University of Birmingham UK, [3] Dept Surgery, Haugesund Hospital Norway, [4] University Hospital Rotterdam The Netherlands, [5] Roswell Park Cancer Institute USA.

The roles of adjuvant chemoradiotherapy and adjuvant chemotherapy in pancreatic cancer remain uncertain. The European Study Group for Pancreatic Cancer's (ESPAC) first study is the largest randomised adjuvant pancreatic trial designed to assess these roles and recruited 541 patients with resected pancreatic ductal adenocarcinoma. Previous studies have been somewhat under powered and often excluded patients with positive resection margins. ESPAC-1 included patients with positive resection margins as recognition of the natural behaviour of the disease and met the target of randomising 285 patients via a 2x2 factorial, with additional patients randomised into chemotherapy only (n=188) or chemoradiotherapy only (n=68) randomisation options. The preliminary results of ESPAC-1, with 314 (58%) deaths and median follow-up of 10 months (inter-quartile range: 1-25) for alive patients, showed no evidence of a survival benefit for chemoradiation but some evidence of benefit for chemotherapy (Lancet 2001; 358: 9293, 1576-1585).

A meta-analysis of the interim data from ESPAC-1 with data from the Gastrointestinal Tumor Study Group (GITSG), the European Organization for Research and Treatment of Cancer (EORTC) and the Norwegian Pancreatic Cancer Trials Group has been carried out to assess world-wide evidence for chemoradiation and chemotherapy. ESPAC-1 dominates the analysis due to it's large size and results confirm no overall benefit for chemoradiation (risk reduction –4%, p=0.71) and a benefit for chemotherapy (risk reduction 35%, p<0.001).

The last ESPAC-1 patient was randomised in April 2000 hence with a minimum follow-up of 2 years final results will be available April 2002. These will be combined with individual patient data from the EORTC and Norwegian groups to allow global investigation of important prognostic variables and confirm treatment effects within specific subgroups of patients with resectable pancreatic adenocarcinoma.

### CT4

### A RANDOMISED TRIAL OF CHOP x 6-8 vs CHOP x 3 + BEAM + ASCT IN 457 PATIENTS WITH POOR PROGNOSIS HISTOLOGICALLY AGGRESSIVE NON-HODGKINS LYMPHOMA

D.C. Linch[1], L. Yung[1], P. Smith[1], K. Maclennan[1], A. Jack[1], B. Hancock[1], D. Cunningham[1], P. Hoskin[1], H. Holte[2], A-M Boesen[2], A. Grigg[3], P. Browett[3] on behalf of [1] UK Lymphoma Group, [2] Nordic Lymphoma Group, [3] Australasian Leukaemia & Lymphoma Group.

Between January 1993 and October 2001 457 patients with newly diagnosed histologically aggressive NHL and poor prognosis disease defined as age-adjusted IPI score of high and high-intermediate risk were entered into this trial. Histology included diffuse large B-cell lymphoma, grade 3 follicular lymphoma, anaplastic large cell lymphoma and peripheral T cell lymphomas. Lymphoblastic lymphomas and Burkitts lymphoma were excluded.

Patients were randomised to receive standard CHOP therapy (6-8 cycles with at least 2 cycles beyond CR) (n=232) or CHOP x 3 followed by BEAM and ASCT (n=225) providing there had been an objective response to CHOP with no progression at any site, and the bone marrow was morphologically clear of lymphoma after 3 cycles of CHOP. Consolidation radiotherapy was at the discretion of the individual centres.

The median age was 48 years (range 16-65) with 278 males and 179 females. The primary endpoint was overall survival (OS) and with a median follow-up of 54 months there is no difference between the two arms ($\chi^2$=0.15, p=0.7). The actuarial overall survival in the CHOP arm was 54% at 5 years and in the CHOP + BEAM arm was 50 % at 5 years. In the CHOP + BEAM arm only 62% of the randomised patients proceeded to BEAM. Reasons for failure to receive BEAM included: inadequate response to CHOP (40%), persisting marrow disease (10%), patient choice (27%), physician choice reflecting the patient's poor general condition (14%) and other (8%). An analysis of progression free survival (PFS) will be presented.
This study shows that high dose therapy after only 3 cycles of CHOP is not of overall benefit in poor risk patients. The lack of adequate response to the initial 3 cycles of CHOP in nearly 20% of patients indicates that future trials must address early intensification of therapy.

### CT5
### MANAGEMENT OF INTERNAL MAMMARY NODES IN SENTINEL NODE BIOPSY

Professor R E Mansel* – Principal Investigator on behalf of the ALMANAC Trialists Group
Department of Surgery, University of Wales College of Medicine, Cardiff

**Introduction:** Sentinel Node Biopsy (SNB) is a guided technique for determining axillary lymph node status of patients with breast cancer. Whilst it is hoped that SNB will eliminate unnecessary surgery for two thirds of breast cancer patients who currently receive standard axillary treatment, either axillary clearance or axillary sampling, to date there has been no detailed evaluation of this new technique in the form of a large randomised trial. One of the issues raised by the SNB technique is the management of the internal mammary nodes.
The ALMANAC trial (Axillary Lymphatic Mapping Against Axillary Nodal Clearance) is a two staged, multi-centre trial comparing SNB with standard axillary treatment in the management of breast cancer. The first stage, now completed, was an Audit stage where surgeons were evaluated on their ability to successfully perform the technique. The second stage, which is currently ongoing, is comparing SNB with standard axillary treatment in terms of arm and axilla morbidity, health economics and quality of life. The Audit stage data is presented here.
**Method:** In the Audit stage, surgeons performed a sentinel node biopsy before going on to perform their standard axillary procedure, either axillary sampling or axillary clearance. The sentinel node was localised using a combined technique of a radioisotope (Technetium 99m) and blue dye (Patent blue V). In accordance with the ALMANAC protocol the patient was either injected with the radioisotope the day before the operation (40MBq) or on the day of operation (20MBq). This was followed by a static lymphoscintiscan at least three hours from the time of injection. The drainage site was recorded and the number of hot nodes noted.
**Results:** In total 29 surgeons took part in the Audit stage and 803 patients were recruited. Eight patients did not receive a scan due to logistical problems. Data was missing on a further 26 patients leaving 769 cases of analysable data. There were 206 cases (27%) where no drainage site was reported on the scan and 563 cases (73%) where drainage was reported. Axillary drainage was reported in 537(70%) cases and internal mammary drainage in 63(8%). Of the 63 patients, only 22(35%) had lymph nodes removed of which 2 'nodes' proved to be fatty tissue. Only 4 patients had internal mammary nodes that were pathologically positive and in 2 of the patients the axillary nodal status was negative. One patient sustained a pneumothorax and one patient suffered bleeding from the internal mammary artery.
**Conclusion:** Removing the internal mammary nodes remains a difficult routine procedure. Due to the relatively small percentage of patients that have positive internal mammary nodes with negative axillary status (ie have a change in stage), we conclude that routine removal of internal mammary nodes will have a minimal effect on the mortality from breast cancer.

### CT6
### THE BIG LUNG TRIAL (BLT): DETERMINING THE VALUE OF CISPLATIN-BASED CHEMOTHERAPY FOR ALL PATIENTS WITH NON-SMALL CELL LUNG CANCER (NSCLC). PRELIMINARY RESULTS IN THE SUPPORTIVE CARE SETTING.

*RJ Stephens[1], JM Brown[2], DJ Fairlamb[3], N Gower[4], L Maslove[5], R Milroy[6], V Napp[2], MD Peake[7], RM Rudd[8], S Spiro[9], *H Thorpe[2], D Waller[7] on behalf of all the participants.
[1]MRC Clinical Trials Unit, London. [2]Northern & Yorkshire Clinical Trials & Research Unit, University of Leeds. [3]Deanesly Centre, New Cross Hospital, Wolverhampton. [4]CRC & UCL Cancer Trials Centre, London. [5]York Health Economics Consortium, University of York. [6]Stobhill NHS Trust, Glasgow. [7]Glenfield Hospital, Leicester. [8]Dept of Medical Oncology, St Bartholomew's Hospital, London. [9]Dept of Thoracic Medicine, The Middlesex Hospital, London.

This large multicentre randomised trial investigated the role of short-term cisplatin-based chemotherapy for all patients with NSCLC. In addition to their primary treatment (surgery, radical radiotherapy or 'best supportive care') patients were randomised to chemotherapy (CT) or no chemotherapy (NoCT). CT could be cisplatin/vindesine (CV), mitomycin/ifosfamide/cisplatin (MIC), mitomycin/ vinblastine/ cisplatin (MVP) or vinorelbine/cisplatin (NP). Recruitment closed on 30 November 2001 with a total of 1394 patients.
**Preliminary results in the supportive care setting**
725 patients were randomised (364 CT, 361 NoCT). Pre-treatment characteristics: median age 65 years, 74% male, 53% squamous cell, 79% WHO PS 0-1. In the CT group: 65% received the prescribed 3 cycles of chemotherapy (48% with no delays or modification), 28% had grade 3/4 toxicity, mainly haematological (12%), nausea/vomiting (4%) and neutropenic fever (2%), and there were 14 (4%) reported treatment-related deaths. There was a significant survival advantage to the CT group: HR 0.77 (95%CI 0.66-0.91), p=0.0015.
Quality of Life (QL) was assessed in a subgroup of 273 patients using patient completed diary cards and the EORTC QLQ-C30 & LC17. There was no evidence of a difference in the pre-defined primary QL endpoint (global health status/QL at 12 weeks) or in any of the secondary endpoints (physical functioning, emotional functioning, fatigue, pain and dyspnoea). Health service costs, estimated in a subgroup of 194 patients using retrospective resource use data collected from hospital records, showed no evidence of a significant offset between the cost of CT and the cost of symptom management.
The preliminary results of this, the largest trial in this setting, confirm the previously reported overall survival benefit with cisplatin-based chemotherapy, as well as the improvement in median survival of approximately 8 weeks. In addition, the sub-studies suggest no evidence of a difference in QL or economic benefit.

### CT7
### RESULTS OF THE SIOP/UKCCSG PNET-3 RANDOMISED STUDY OF PRE-RADIOTHERAPY CHEMOTHERAPY COMPARED WITH RADIOTHERAPY ALONE IN M0/M1 MEDULLOBLASTOMA

*Taylor R[1], Bailey C[2], Robinson K[3], Weston C[3], Lucraft H[4], Gilbertson R[4], Lashford L[3].
1 Cookridge Hospital, Leeds 2 University of Leeds, 3 United Kingdom Children's Cancer Study Group (UKCCSG), Leicester, Royal Victoria Infirmary, Newcastle.

**Aim of the Study**
The was to determine whether four cycles of chemotherapy given after surgery and before radiotherapy (RT) would improve the outcome for patients with M0-1 medulloblastoma compared with RT alone.
**Patients and Methods**
Patients aged 3-16 inclusive were randomised to either chemotherapy (vincristine 1.5 mg/m$^2$ weekly for three weeks, etoposide 100mg/m$^2$ daily for 3 days and carboplatin 500 mg/m$^2$ daily for 2 days alternating with cyclophosphamide 1.5 g/m$^2$) followed by craniospinal RT 35 Gy in 21 fractions with a boost of 20 Gy in 12 fractions to the posterior fossa, or RT alone.
**Results**
217 patients were randomised and 179 eligible for analysis (chemotherapy + RT: 90, RT alone: 89). Median age was 7.67 years. Median follow-up was 4.71 years. For all eligible patients overall survival (OS) was 78.9% at 3 years and 71.4% at 5 years. Event free survival (EFS) was 71.5% at 3 years and 66.7% at 5 years. A significant difference in EFS was demonstrated for chemotherapy + RT compared with RT alone. For chemotherapy + RT, EFS was 78.7% at 3 years and 73.4% at 5 years compared with 64.2% at 3 years and 60.0% at 5 years for RT alone (p=0.0419). There was no statistically significant difference in 3-year and 5-year OS between the two arms (82.1% and 76.1% for Chemotherapy + RT, compared with 75.8% and 66.5% for RT alone, p = 0.1662). For patients who had undergone a total resection EFS was significantly better with chemotherapy + RT than with RT alone (p=0.0346), but not for patients who had undergone a less than total resection (p=0.4835).

© 2002 Cancer Research UK

There was a significantly better EFS (p=0.0184) for patients completing RT within 50 days compared with those taking more than 50 days to complete. Multivariate analysis identified extent of surgical resection (p=0.0398), use of chemotherapy (p=0.0228) and time to complete RT (0.0056) as having a significant impact on EFS.

**Conclusions**

This is the first multi-centre, randomised study to demonstrate an improved EFS for patients with medulloblastoma treated with pre-RT chemotherapy compared with RT alone. The importance of avoiding gaps in RT schedules has been confirmed.

### CT8

### THE EMI STUDY: A REGIONAL FEASIBILITY STUDY FOR A RANDOMISED TRIAL OF ADJUVANT CHEMOTHERAPY FOLLOWING DEFINITIVE TREATMENT FOR TRANSITIONAL CELL CANCER (TCC) OF THE BLADDER.

*MG Leahy[1], J Brown[2], WG Jones[3], J Kelly[4], DE Neal[4], S Prescott[5], T Roberts[6], P Selby[7]
[1] University of Leeds, School of Medicine. [2] Northern and Yorkshire Clinical Trials and Research Unit. [3] Leeds Cancer Centre. [4] University of Newcastle. [5] Leeds Teaching Hospitals NHS Trust. [6] Northern Centre for Cancer Treatment. [7] Cancer Research UK Clinical Centre in Leeds.

Introduction: Cancer networks have the potential to make a powerful contribution to cancer research through collaborative studies. We conducted a feasibility study in the Northern and Yorkshire Region that attempted to recruit all eligible patients (pts.) into a randomised trial of adjuvant chemotherapy following radical local treatment for muscle invasive TCC of the bladder.

Patients and Methods: The protocol was approved by Northern and Yorkshire Multicentre Research Ethics Committee. The target was to randomise 60 pts. within 2 years. Consenting pts. were registered at diagnosis. Following primary therapy (cystectectomy [CYST] or radical radiotherapy [RRT]), pts. were assessed for fitness to start 3 cycles of MVAC chemotherapy within 12 weeks. If suitable, they were given further information and randomised if they consented.

Result: The trial was activated in 20 hospitals with enthusiastic support. 354 pts. were registered. 21% were not suitable for radical primary therapy due to metastatic disease or co-morbidity. Of the remainder, 50% had CYST and 50% had RRT. After CYST/RRT, 67% / 81% were medically unfit for chemotherapy. Of the 15% eligible for randomisation, 75% declined. The final number of patients randomised was 6.

Conclusion: This study demonstrates that population-based cancer research within the NHS is feasible. In the patient group studied there was a higher incidence of co-morbidity than expected and many pts. declined randomisation. This experience is relevant to the development of the new National Cancer Research Network. The difficulty in recruitment in this area supports the recent decision by co-operative groups to collaborate in a international intergroup study to address the issue of adjuvant chemotherapy for bladder cancer.

© 2002 Cancer Research UK

